# Detection of H5N1-Related PB1 Sequences in a Low Pathogenic H11N2 Virus from South American Migratory Shorebirds

**DOI:** 10.3390/v18070710

**Published:** 2026-06-27

**Authors:** Jansen de Araujo, Helena Lage Ferreira, Thomas P. Fabrizio, Luciano Matsumiya Thomazelli, David Walker, Tatiana Ometto, Giovana Santos Caleiro, Desyrée Yumiko Sadoyama Rangel Ozaki, Nicole Almeida dos Reis, Gustavo Oliveira Fenner, Fernanda Panicio Vizu, Antônio Coimbra de Brum, Mateus Luís Haas, Júlia Victória Grohmann Finger, Maria Virginia Petry, Victória Deecken Becker, Douglas Ribeiro da Silva, Pedro Henrique de Oliveira Hoffmann, Isabele Colla Lazzari Royes, João Renato R. Pinho, Deyvid Amgarten, Erick G. Dorlass, Ana L. Boechat Borges, Fernanda de Mello Malta, Danielle Bruna L. Oliveira, Alessandra Greatti, Robert G. Webster, Richard J. Webby, Clarice Weis Arns, Edison L. Durigon

**Affiliations:** 1 Laboratório de Pesquisa em vírus Emergentes and Laboratório de Virologia Clínica e Molecular at Biomedical Science Institute, University of São Paulo, Av. Prof. Lineu Prestes 1374, São Paulo 05508-000, SP, Brazildesyumi.walker@gmail.com (D.Y.S.R.O.);; 2 Graduate Program in Epidemiology and One Health, Veterinary Medicine and Animal Science School, University of São Paulo, Av. Prof. Orlando Marques de Paiva, 87, Butantã, São Paulo 05508-270, SP, Brazil; 3 Department of Veterinary Medicine, FZEA-USP, University of São Paulo, 225 Av Duque de Caxias Norte, Pirassununga 13635-900, SP, Brazil; 4 Department of Host-Microbe Interactions, St. Jude Children’s Research Hospital, Memphis, TN, USA; 5 CNPEM-Centro Nacional de Pesquisa em Energia e Materiais, Campinas, SP, Brazil; 6 Laboratório de Ornitologia e Animais Marinhos, Universidade do Vale do Rio do Sinos (UNISINOS), São Leopoldo, RS, Brazil; 7 Programa de Pós-graduação em Biologia de Ambientes Aquáticos Continentais (PPG-BAC), Instituto de Ciências Biológicas, Universidade Federal do Rio Grande—FURG, Avenida Itália, Km 8, Rio Grande 96203-900, RS, Brazil; 8 Preservas-Núcleo de Conservação e Reabilitação de Animais Silvestres da Faculdade de Veterinária da Universidade Federal do Rio Grande do Sul–UFRGS, Porto Alegre, RS, Brazil; 9 Hospital Israelita Albert Einstein, Av. Albert Einstein, 627/701, São Paulo 05652-900, SP, Brazil; joao.pinho@einstein.br (J.R.R.P.);; 10 Department of Genetics, Evolution, Microbiology and Immunology, Institute of Biology, University of Campinas—UNICAMP, P.O. Box 6109, Campinas 13083-970, SP, Brazil; 11 Institut Pasteur of São Paulo, São Paulo, SP, Brazil

**Keywords:** genetic reassortment, migratory shorebirds, H5N1 clade 2.3.4.4b, avian influenza surveillance, South America

## Abstract

Highly pathogenic avian influenza (HPAI) A(H5N1) viruses of clade 2.3.4.4b have recently spread across the Americas, prompting intensified surveillance efforts in Brazil aimed at early detection in wild birds. As part of these efforts, we identified a low pathogenic avian influenza A(H11N2) virus in a white-rumped sandpiper (*Calidris fuscicollis*) sampled at Lagoa do Peixe National Park (PNLP) in southern Brazil. Whole-genome sequencing revealed that seven of the eight gene segments shared high nucleotide similarity (approximately 98.8%) with viruses previously detected in shorebirds from Delaware Bay, North America. In contrast, the PB1 segment showed high nucleotide similarity (approximately 99%) to the PB1 lineage associated with clade 2.3.4.4b A(H5N1) genotype B3.2 viruses circulating in the Americas. Phylogenetic, nucleotide identity, and molecular clock analyses indicated that this lineage shares a recent common ancestor with North American LPAI viruses and was subsequently detected in distinct viral genetic backgrounds. Although no HPAI virus was identified in this study, the presence of a PB1 segment related to H5N1-associated lineages suggests that genetic components linked to these viruses were circulating among low pathogenic avian influenza viruses in South America. These findings highlight the importance of continued surveillance in migratory bird populations to improve understanding of avian influenza virus diversity and support epidemiological monitoring.

## 1. Introduction

Influenza A is a prime example of a viral disease where sustained virus evolution is responsible for annual epidemics and intermittent pandemics in humans. The diversity of influenza A viruses (IAVs) in aquatic birds is a driver for the emergence of pandemic influenza in humans and infections in wild mammals and birds [1]. The evolution of IAV is most noticeably seen in the surface glycoproteins, but it also affects the other six genomic segments. IAV variability arises from molecular changes that accumulate through multiple mechanisms, including “genetic drift” and “genetic shift” that can occur in humans, pigs, horses, and birds [2]. South America has historically been underrepresented in genomic surveillance efforts, resulting in limited understanding of local avian influenza viruses (AIV) diversity and evolutionary dynamics [3,4]. Nevertheless, increasing evidence indicates that the continent harbors distinct viral lineages maintained within regional bird populations, while also receiving gene flow from North American viruses during migratory periods [5]. Despite years of global dissemination and the well-recognized threat posed by HPAI H5 viruses, clade 2.3.4.4b represents the first confirmed establishment of these viruses in South America, marking a pivotal epidemiological shift. This emergence is supported by growing regional evidence from Peru, Chile [6], Argentina [7,8], Uruguay [9], and Brazil [10], with outbreaks consistently associated with the B3.2 genotype, underscoring both the continental-scale spread of a common viral lineage and the importance of situating local findings within this rapidly expanding South American context.

Since late 2022, H5N1 has been reported across these countries, affecting wild birds, poultry, and both marine and terrestrial mammals, with multiple mortality events and two confirmed human infections [11,12,13,14,15].

In Brazil, the first detection occurred in May 2023 in a Cabot’s tern [16]. Genomic analyses indicate that circulating viruses are predominantly of the B3.2 genotype, comprising Eurasian-origin H5 segments reassorted with North American low pathogenic avian influenza (LPAI) gene segments [17,18]. This expanding body of evidence highlights the rapid dissemination of HPAI across the continent and underscores the importance of understanding how local LPAI viruses interact with newly introduced lineages. In addition to this urgent epizootic, Brazil is the biggest poultry meat exporter and the third largest producer of poultry meat worldwide, which reinforces the need for surveillance in all national territory [10,19]. To reinforce surveillance activities and fill gaps, initiatives such as the PREVIR/MCTI Network were established to monitor emerging viruses in wild animals across multiple Brazilian biomes [20,21]. As part of these efforts, active surveillance of migratory birds has been intensified to enable early detection of avian influenza viruses, particularly in ecologically relevant sites that connect intercontinental flyways. Within this surveillance framework, we report the detection and genomic characterization of a low pathogenic avian influenza A(H11N2) virus identified in a migratory shorebird sampled at Lagoa do Peixe National Park (PNLP), a key stopover site in southern Brazil [22]. Phylogenetic analyses indicate that this virus is a reassortant containing gene segments associated with North American lineages, including a PB1 segment closely related to the lineage later identified in HPAI H5N1 viruses of the B3.2 genotype. Molecular clock analyses further suggest that the PB1 segments detected in the PNLP H11N2 and H5N1 B3.2 viruses share a recent common ancestor derived from North American LPAI lineages. These findings provide the first evidence of an H11 subtype at the PNLP site, with this genomic constellation, and expand current knowledge of AIV diversity in South America during the emergence of H5N1 in South America and Brazil.

## 2. Materials and Methods

### 2.1. Sample Collection

**Figure 1:**
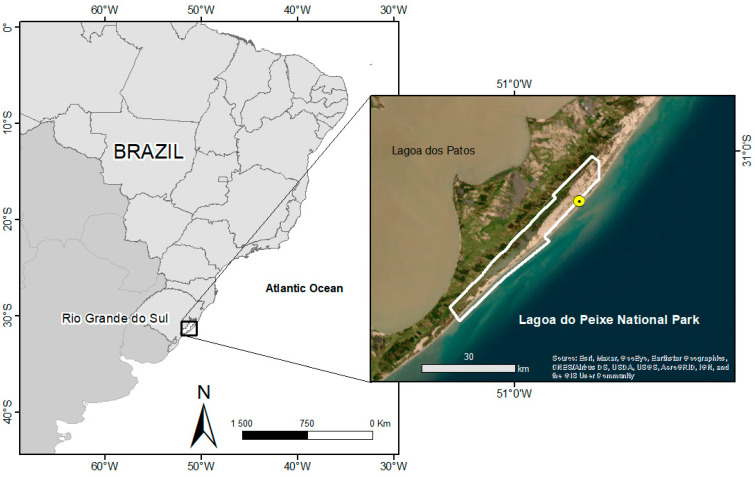
Location of the sampling area: mid-coast of Rio Grande do Sul State, facing Lagoa do Peixe National Park (PNLP). The yellow circle indicates the location where shorebirds were trapped in January 2023.

Cloacal and tracheal samples were collected at a conservation unit in the extreme South of Brazil (Lagoa do Peixe National Park—PNLP: 31°16′44.4″ S 50°56′09.9″ W and the mid-coast of Rio Grande do Sul State) between the 3rd and 8th of January 2023 (Figure 1).

**Figure 2:**
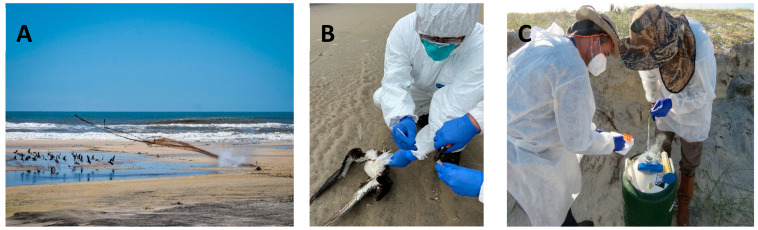
Sample collection: (**A**) Trapping shorebirds with a cannon-net, (**B**) Swabs sampling from animals, and (**C**) Storage of samples in liquid nitrogen.

Shorebirds were trapped with a cannon-net that was handled by experienced researchers (Figure 2). Each sample was stored in a cryotube containing Viral Transport Medium (VTM), composed of PBS supplemented with antimicrobial agents (200 U/mL penicillin G, 200 U/mL streptomycin, 25 μg/mL fungizone, and 6 μg/mL gentamycin) and 10% glycerol. Following collection at PNLP, samples were stored in a liquid nitrogen dry shipper and transported to the University of São Paulo for the sampling process.

### 2.2. AIV Detection

Samples were received and processed at the Biomedical Institute of the University of São Paulo (ICB-II-USP). RNA was extracted using the MagMax TM-96 RNA Isolation Kit (Ambion, Austin, TX, USA), following the manufacturer’s instructions. The RNA was then screened for the presence of the AIV matrix gene using a one-step real-time reverse transcriptase (RT)-PCR and AIV-M TaqMan Kit (Applied Biosystems, Foster City, CA, USA), as previously described.

### 2.3. Sequencing and Phylogenetic Analysis

The positive sample was subjected to next-generation sequencing (NGS) using sample enrichment according to the previously described methodology [23,24]. Obtained sequences were compared with the NCBI database through BLAST searches. Briefly, samples were firstly isolated and purified with RNA Clean & Concentrator kit (Zymo Research, Irvine, CA, USA), followed by random amplification and sequencing library amplification with NextTera XT kit (Illumina, San Diego, CA, USA). Resulting libraries were submitted to sequencing with the NextSeq 550 platform. Generated reads were submitted to the VarsMetagen platform were Virome pipeline was executed. Reads were filtered and mapped to the host genome (*Gallus gallus* assembly: GCF_016699485.2) with the BWA tool. Unmapped reads were gathered and submitted to the first round of identification with Kraken2 and then assembled with SPADES and submitted to the second round of identification.

Phylogenetic relationships were inferred independently for each gene segment using a maximum likelihood (ML) approach. Sequence alignments were constructed using reference datasets retrieved from the GISAID database, including representative avian influenza virus strains from North and South America. Each genomic segment was subjected to BLAST search against the GISAID EpiFlu database. A total of 865 sequences were selected for the PB1 segment, and 200 sequences for the remaining segments for multiple sequence alignment construction. Nucleotide sequence alignment was performed with MAFFT using default parameters; each alignment was manually checked with Aliview. Phylogenetic trees were created by Maximum Likelihood analysis using IQ-TREE. The best-fit nucleotide substitution model for each dataset was selected using the ModelFinder parameter. Each tree was constructed with 1000 replicas for statistical support. Branch support was evaluated using ultrafast bootstrap resampling, and only nodes with support values above 70 were considered for interpretation. To investigate genomic composition and phylogenetic incongruences among gene segments, genome segments were concatenated following segment order (PB2, PB1, PA, HA, NP, NA, MP, and NS). The highest identity H11N2 and H5N1 sequences were selected after BLAST searches against the GISAID EpiFlu database. The H11N2 reference was selected based on HA results, whereas the H5N1 reference was selected based on PB1 results. Pairwise distances were calculated using Recan [25], with sliding window and step sizes of 200 and 50 nucleotides, respectively, to evaluate segment-specific nucleotide identity patterns across the concatenated genome. Phylogenetic topologies were also compared across all eight influenza A virus gene segments. Segment-specific incongruences in clustering patterns were interpreted as signatures of distinct evolutionary histories. Phylogenetic discordance, together with high nucleotide identity, Recan profiles, and strong bootstrap support (≥90%), was evaluated as evidence compatible with reassortment or differential ancestry among viral gene segments.

Molecular clock analyses were performed to estimate the time to the most recent common ancestor (tMRCA) between the PNLP virus and related H5N1 sequences. A representative dataset of 43 sequences was selected from the maximum likelihood phylogenies, and a root-to-tip regression was performed using TempEst v1.5.3 to assess temporal signal. The constructed dataset included PNLP viruses and closely related sequences of the H5N1 B3.2 genotype from North and South America (2022–2023), as well as previously circulating LPAI viruses. Time-scaled phylogenies were reconstructed in BEASTX v10.5.1 under the SDR06 substitution model with substitution rate estimated during the analysis. A Coalescent Constant Population prior, and a GMRF Bayesian Skyride model were evaluated under an uncorrelated relaxed molecular clock. Both demographic models were compared to assess the consistency of the estimated tMRCA values. [26]. Markov Chain Monte Carlo (MCMC) runs of 100 million steps were performed two times, with parameters sampled at every 10.000 steps, logs and treefiles were combined with LogCombiner v10.5.1. Convergence and mixing were assessed in Tracer v1.7, and all relevant parameters showed ESS values greater than 200. Maximum clade credibility (MCC) trees were generated using TreeAnnotator and visualized in FigTree.

## 3. Results

**Figure 3:**
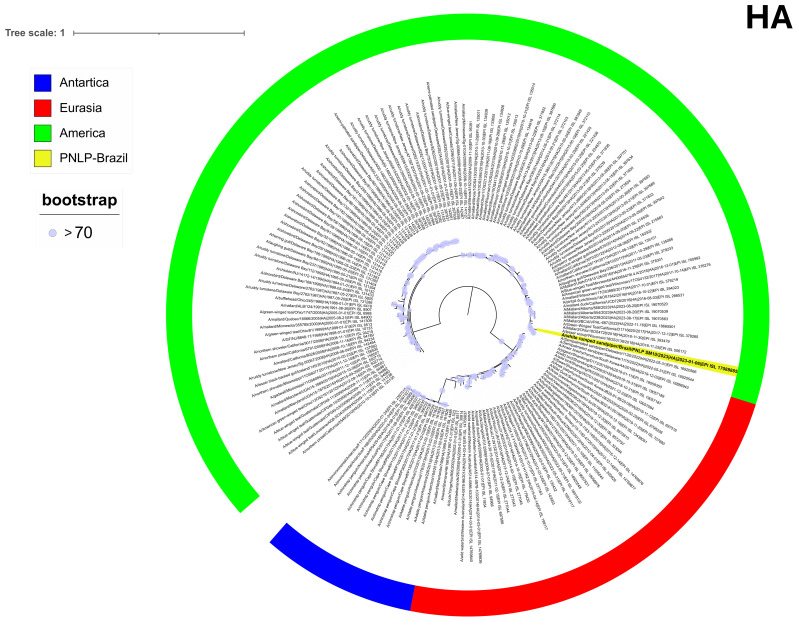
Circular phylogenetic tree of the gene encoding the hemagglutinin (HA) gene of avian influenza viruses H11N2. The PNLP sequence is marked in yellow from *Calidris fuscicollis* (A/White-rumped sandpiper/RS-Brazil/A10/2023). A total of 164 sequences were used for the analysis, with an alignment of 1698 nucleotides in length. Branches with a bootstrap value of 70 are marked with a circle. IQTree Model Finder used the GTR + F + I + G4 substitution model according to BIC values.

**Figure 4:**
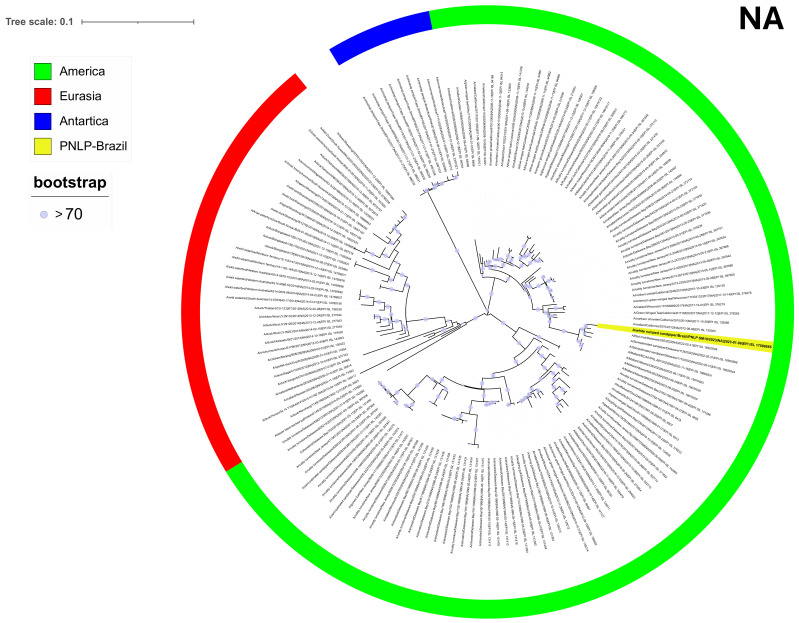
Circular phylogenetic tree of the gene encoding the neuraminidase (NA) gene of avian influenza viruses. The PNLP sequence is marked in yellow from *Calidris fuscicollis* (A/White-rumped sandpiper/RS-Brazil/A10/2023). A total of 158 sequences were used for the analysis, with an alignment of 1410 nucleotides in length. Branches with bootstrap values over 70 are marked with a circle. IQTree Model Finder used the TVM + F + I + G4 substitution model according to BIC values.

A total of 40 tracheal and oral swab samples were collected from wild birds, including five migratory species and one resident species, the kelp gull *Larus dominicanus* (n = 1). The sampled migratory species included American golden-plovers (*Pluvialis dominica*, n = 1), greater yellowlegs (*Tringa melanoleuca*, n = 2), common terns (*Sterna hirundo*, n = 1), white-rumped sandpipers (*Calidris fuscicollis*, n = 18), and sanderlings (*Calidris alba*, n = 17). Among these animals, two white-rumped sandpipers and the kelp gull exhibited neurological signs, including prostration, leg paralysis, and lack of response to stimulation. However, only one of the symptomatic white-rumped sandpipers tested positive for avian influenza virus (AIV) by RT-qPCR (Ct = 27). All other samples, including the kelp gull, were negative. Given the lack of consistent detection of AIV in symptomatic birds, these findings do not directly support a causal association between the observed clinical signs and AIV infection. Whole-genome sequencing was successfully performed directly from the positive swab sample, and the virus was identified as a low pathogenic avian influenza A(H11N2). Phylogenetic analyses showed that seven of the eight gene segments clustered with North American lineage AIVs, particularly viruses detected in *Calidris pusilla* from Delaware Bay, United States, in 2022 (Figure 3, Figure 4 and Figures S1–S5, Table S2).

**Figure 5:**
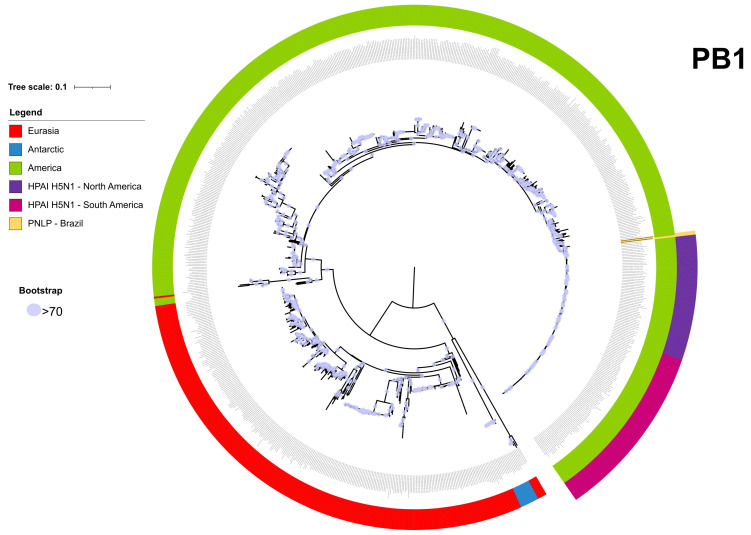
Circular phylogenetic tree of the PB1 segment. The PNLP sequence is marked in yellow from *Calidris fuscicollis* (A/White-rumped sandpiper/RS-Brazil/A10/2023). HPAI H5N1 sequences are highlighted with a purple sector. A total of 865 sequences of different Influenza A subtypes were used for the analysis, with an alignment of 2274 nucleotides in length. Branches with bootstrap values over 70 are marked with a circle. IQTree Model Finder used the GTR + F + I + I + R5 substitution model according to BIC values.

**Figure 6:**
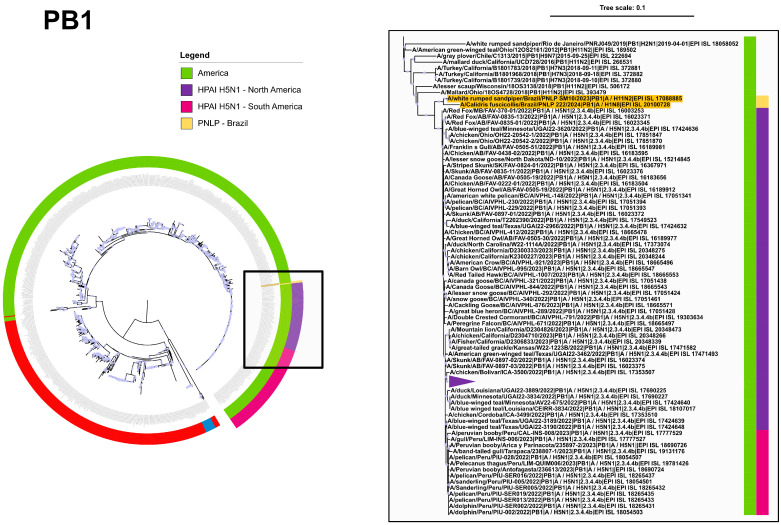
Rectangular phylogenetic tree of PB1 segment. Cropped segments are highlighted for a better view. The PNLP sequence is marked in red from *Calidris fuscicollis* (A/White-rumped sandpiper/RS-Brazil/A10/2023) and all HPAI (H5N1) in blue. A total of 865 sequences were used for this analysis, with an alignment of 2274 nucleotides in length. Branches with bootstrap values over 70 are marked with a circle. IQTree Model Finder used the GTR + F + I + I + R5 substitution model according to BIC values.

In contrast, the PB1 gene segment grouped within a clade containing sequences from LPAI and HPAI A(H5N1) viruses of clade 2.3.4.4b circulating in the Americas, including strains from the U.S, Chile, Peru, and Ecuador (Figure 5 and Figure 6). Phylogenetic analysis of the PB1 segment indicates that the clusters in proximity to a South American H1N8 strain collected in 2024, share high nucleotide identity (98%). The PB1 segment of the detected virus differed by 27–43 nucleotides and 3 main amino acid substitutions from its closest relatives (Table 1 and Table S2). The analyzed dataset includes sequences from North Dakota (USA), Mexico, Ecuador (FBC/FBT), and Chile. The focal sequence shows approximately 27 nucleotide differences relative to the North Dakota strain and 34–43 differences compared to Mexico, Chile, and Ecuadorian H5N1 viruses, supporting a close relationship with evidence of moderate genetic drift within this lineage (Table 1).

**Figure 7:**
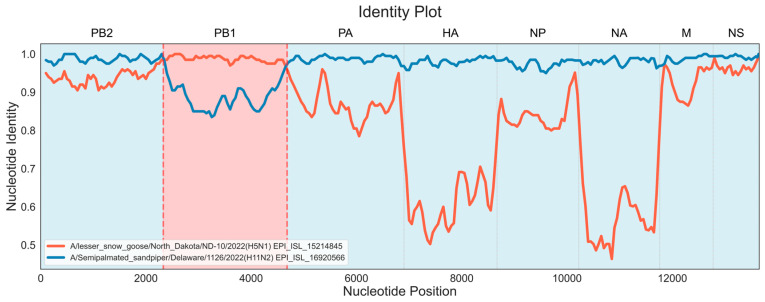
Nucleotide identity plot. Obtained A/white_rumped_sandpiper/Brazil/PNLP_SM10/2023(H11N2) relative nucleotide identity with H5N1 (EPI_ISL_15214845, red) and H11N2 (EPI_ISL_16920566, blue). Genome segments were concatenated (segments 1 to 8), and pairwise distances were calculated with Recan. Although 7 of the 8 segments showed higher identity with H11N2, the PB1 segment presented high identity with H5N1 (red dashed lines, positions 2329 to 4682).

**Figure 8:**
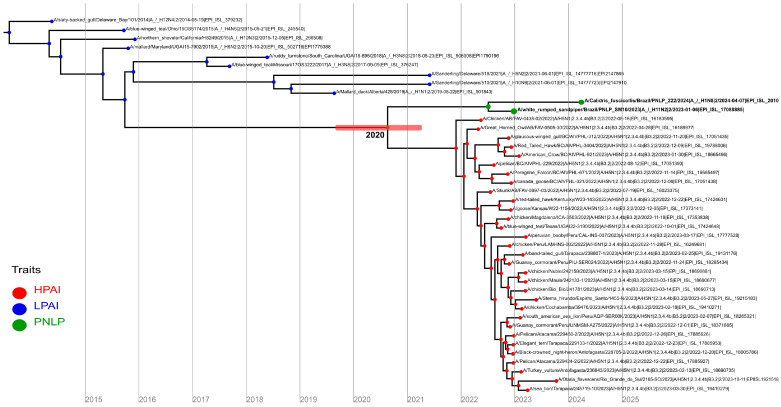
Time-scaled maximum clade credibility phylogeny of the PB1 segment inferred in BEAST under the SDR06 substitution model, a Coalescent Constant Population prior, and an uncorrelated relaxed molecular clock. The PNLP H11N2/H1N8 viruses are highlighted in green, LPAI viruses in blue, and HPAI H5N1 viruses in red.

Nucleotide identity profiles were evaluated across the concatenated genome using Recan (Figure 7). Seven of the eight genomic segments showed higher nucleotide identity with the North American H11N2 reference virus (EPI_ISL_16920566). In contrast, the PB1 segment showed higher nucleotide identity with the H5N1-associated PB1 lineage. Molecular clock analyses estimated the tMRCA of the PNLP PB1 lineage and related H5N1-associated PB1 sequences at approximately 2020 (Figure 8/95% HPD: 2019–2021), predating the introduction of H5N1 in North America, which occurred at late 2022 [17,18].

The surface glycoproteins did not cluster with previously described Brazilian H11 or N2 viruses, including those identified in shorebirds of the Amazon region, PNLP, or Rio de Janeiro [2,22,27]. Overall, these results indicate a predominantly North American genomic composition for the PNLP virus.

## 4. Discussion

The phylogenetic profile of the detected virus indicates that similar subtypes may be introduced into South America multiple times from distinct lineages [28]. The genomic composition of the PNLP H11N2 virus differs from previously reported South American H11 and N2 [4,22,27]. HA, NA, and all internal segments clustered with North American H11N2 subtypes from Delaware, 2022 (S1–S6), except the PB1 segment grouped within a broader H5N1 clade. This specific constellation of segments, together with the first detection of an H11 HA type virus at PNLP, highlights a distinct viral genotype not previously reported in the region. Overall, this pattern indicates a reassortant virus with a predominantly North American genomic composition rather than a local subtype. Together, these findings expand the currently limited knowledge of AIV diversity in Brazil and South America, adding evidence from an underrepresented region and highlighting the role of migratory birds in introducing distinct viral lineages across the continent [5].

Although based on a single isolate and therefore not representative of regional viral circulation, this finding is noteworthy because it was detected during a period when most avian influenza reports in South America were associated with the emergence and spread of HPAI viruses [16,18]. Notably, the H11N2 virus described here was among the few LPAI viruses reported in Brazil and South America during 2023, together with the H5N2 virus detected in the Pantanal and an H6N2 virus identified in Brazil [29,30]. These findings highlight the diversity of influenza A viruses circulating in migratory bird populations during this epidemiological period.

White-rumped sandpipers migrate from the Canadian Arctic, where they breed during the Northern Hemisphere winter, to South America, where they stay during summer in the southern continent [31,32], where other pathogens were already described, including coronaviruses detected along the mid-coast of Rio Grande do Sul [21].

The extensive migratory range of these species highlights their role as a transcontinental vector for the dissemination of avian viruses across the Americas. At the time of sampling in January 2023, South America was experiencing the early stages of H5N1 clade 2.3.4.4b dissemination, with the first mass mortality events being reported in Peru and Chile [33,34], but not in Brazil. In this context, the identification of a PB1 gene with high nucleotide similarity to H5N1-associated sequences requires careful interpretation.

Although the PB1 segment clusters with H5N1-associated lineages, our data do not support a recent reassortment event involving currently circulating HPAI H5N1 viruses and the PNLP H11N2 strain. Instead, this pattern reflects a shared ancestry with the broader H5N1 B3.2-associated lineage, derived from North American LPAI viruses and classified as genotype Am. 1.2 [17,35]. Consistent with this interpretation, nucleotide identity analyses indicated that only the PB1 segment diverged from the genomic background of the PNLP virus, while molecular clock analyses estimated the tMRCA of this lineage at approximately 2020 (Figure 8). Together, these findings support the hypothesis that the PB1 segment originated from a North American LPAI lineage and was subsequently detected in distinct viral genetic backgrounds, including the PNLP virus and H5N1 B3.2 viruses.

Due to the high amino acid similarity observed between H11N2 and H5N1 PB1 sequences, the main amino acid differences between these groups were compared (Supplementary Materials). Few substitutions were identified, including E264D, K429R, and L744M, which were conserved among most H5N1 sequences (>98%) but absent from the H11N2 dataset. Although these substitutions have been previously reported in avian influenza viruses, their biological significance in the context of the present study remains unclear. In contrast, known markers such as D3V and 66S (PB1-F2) were detected in both groups, consistent with standing genetic variation rather than subtype-specific evolution [36,37].

Notably, the detection of a gene closely related to those found in H5N1 viruses before the first reported cases in Brazil (May 2023) raises important considerations for surveillance networks and, ultimately, for poultry production systems, particularly in a country that plays a major role in global poultry production. Although the virus described here was detected in wild migratory birds and does not represent a direct threat to poultry production, its detection highlights the importance of surveillance in regions where wildlife and domestic animal populations may interact. Notably, Rio Grande do Sul was later the site of the first HPAI H5N1 outbreak reported in a commercial poultry farm in Brazil, which occurred in Montenegro, approximately 300 km from the PNLP sampling area [19].

Even in the absence of direct detection of HPAI viruses, the circulation of genetically related segments highlights the potential for viral introduction and reinforces the importance of continuous surveillance at the wildlife–domestic interface. The detection of genetically related viral segments during periods of HPAI emergence may improve risk assessment and support preparedness strategies to mitigate economic and sanitary impacts.

Some limitations should be acknowledged. The detection of a single positive sample and the limited sampling effort provide only a snapshot of the ecological context before H5N1 circulation in Brazil. While phylogenetic, nucleotide identity, and molecular clock analyses provide convergent evidence for a reassortment event involving a North American LPAI-derived PB1 lineage, the precise temporal, geographic, and host related circumstances under which this reassortment occurred remain unresolved. Future studies integrating expanded genomic surveillance across migratory and resident bird populations will be essential to reconstruct the evolutionary history of this lineage with greater confidence. In addition, the lack of consistent AIV detection among symptomatic birds and the absence of pathological investigations limit the ability to establish a definitive association between the observed clinical signs and avian influenza virus infection. Alternative causes of mortality should also be considered, including other microbial infections such as type C botulism, which has previously been documented in wild birds from the same southern region in Brazil [38].

## 5. Conclusions

In conclusion, the identification of an LPAI A(H11N2) virus in a migratory white-rumped sandpiper expands the current understanding of avian influenza diversity in Brazil and South America. The detection of a PB1 segment phylogenetically related to clade 2.3.4.4b A(H5N1) genotype B3.2 viruses is particularly noteworthy. Our analyses indicate that both the PNLP H11N2 virus and H5N1 B3.2 viruses share a recent common PB1 ancestor derived from North American LPAI lineages, predating the introduction of HPAI viruses into South America. Although based on a single detection, this finding was obtained during the period of H5N1 circulation and expansion in South America and expands our understanding of LPAI diversity in migratory bird populations and highlights the importance of continued surveillance of LPAI viruses and underrepresented regions, such as South America, particularly during periods of HPAI emergence and expansion.

## Figures and Tables

**Table 1:** Alignment focused on the coding region of the PB1 segment from avian influenza virus (AIV) identified in *Calidris fuscicollis*, compared to H5N1 sequences from the closest phylogenetic branch. Pairwise comparisons are shown as a heatmap, with percentage identity values (%) displayed in the lower triangle and the number of nucleotide differences in the upper triangle. Warmer colors (red tones) indicate a higher number of nucleotide differences and greater genetic divergence, whereas cooler colors (blue tones) reflect higher similarity and fewer differences.

		1	2	3	4	5	6	7	8
**A/White-rumped sandpiper/RS-Brazil/A10/2023**	**1**		27	36	43	36	38	34	35
A/snow goose/North Dakota/ND-10/2022-PB1	**2**	98.81		13	20	13	15	11	14
A_Falco_rusticolus_EdoMex_CPA-19638-22_2022-PB1	**3**	98.42	99.43		23	16	18	12	15
Ecuador-FBT3-PB1	**4**	**98.11**	99.12	98.99		11	12	21	24
Ecuador-FBC2-PB1	**5**	98.42	99.43	99.30	99.52		7	14	17
Ecuador-FBC4-PB1	**6**	98.33	99.34	99.21	99.47	99.69		16	19
A/grey gull/Chile/61947/2022-PB1	**7**	98.50	99.52	99.47	99.08	99.38	99.30		5
A/black skimmer/Chile/61962/2022-PB1	**8**	98.46	99.38	99.34	98.94	99.25	99.16	99.78	

## Data Availability

The data supporting the findings of this study are available in the National Center for Biotechnology Information (NCBI) at https://www.ncbi.nlm.nih.gov, accessed on 1 March 2023. Nucleotide sequence data reported are available in the GenBank databases under the accession numbers OQ533061 to OQ533068 for the A/white_rumped_sandpiper/Brazil/PNLP_SM10/2023(H11N2).

## Supplementary Materials

The following supporting information can be downloaded at: https://www.mdpi.com/article/10.3390/v18070710/s1, Figures S1–S5, and Table titles: Figure S1: Circular phylogenetic tree of PB2 gene of avian H11N2. PNLP sequence is marked in yellow. A total of 160 sequences were used for the analysis, with an alignment of 2280 nucleotides of length. Branches with bootstrap value over 70 are marked with a circle. IQTree Model Finder used GTR + F + I + G4 substitution model according to BIC value. Figure S2: Circular phylogenetic tree of PA gene of avian H11N2. PNLP sequence is marked in yellow. A total of 159 sequences were used for the analysis, with an alignment of 2151 nucleotides of length. Branches with bootstrap value over 70 are marked with a circle. IQTree Model Finder used GTR + F + I + G4 substitution model according to BIC value. Figure S3: Circular phylogenetic tree of NP gene of avian H11N2. PNLP sequence is marked in yellow. A total of 168 sequences were used for the analysis, with an alignment of 1497 nucleotides of length. Branches with bootstrap value over 70 are marked with a circle. IQTree Model Finder used GTR + F + I + G4 substitution model according to BIC values. Figure S4: Circular phylogenetic tree of MP gene of avian H11N2. PNLP sequence is marked in yellow. A total of 164 sequences were used for the analysis, with an alignment of 982 nucleotides of length. Branches with bootstrap value over 70 are marked with a circle. IQTree Model Finder used TIM2e + I + G4 substitution model according to BIC value. Figure S5: Circular phylogenetic tree of the NS gene of avian H11N2. PNLP sequence is marked in yellow. Co. A total of 162 sequences were used for the analysis, with an alignment of 839 nucleotides of length. Branches with bootstrap value over 70 are marked with a circle. IQTree Model Finder used the TVM + F + I + G4 substitution model according to BIC values. Table S1: Genomic characterization of viral segments and closest relatives. Table S2: Comparison of Amino acid substitutions in the PB1 segment of representative H5N1 (HPAI) viruses relative to A/white_rumped_sandpiper/Brazil/PNLP_SM10/2023 (H11N2).
