# Supplementary material for: Detection of H5N1-Related PB1 Sequences in a Low Pathogenic H11N2 Virus from South American Migratory Shorebirds
Source: Viruses. 2026 Jun 27;18(7):710. doi: 10.3390/v18070710 (PMC13431619; doi:10.3390/v18070710)
Supplement: Supplementary file 1 [file viruses-18-00710-s001.zip › viruses-4317979-Table S1.pdf]

Table S1: Genomic characterization of viral segments and closest relatives

Supplementary reference	Segment	Closest Relative Virus	Subtype	Nucleotide Identity (%)	Bootstrap Support (%)	GISAID Accession
S1	PB2	*A/Semipalmated_sandpiper/Delaware/1115/2022*	H11N2	98.73	100	EPI_ISL_16920544
S2	PA	*A/Semipalmated_sandpiper/Delaware/1115/2022*	H11N2	98.73	100	EPI_ISL_16920544
S3	NP	*A/Semipalmated_sandpiper/Delaware/1115/2022*	H11N2	97.47	100	EPI_ISL_16920544
S4	M	*A/Semipalmated_sandpiper/Delaware/1115/2022*	H11N2	98.80	100	EPI_ISL_16920544
S5	NS	*A/Semipalmated_sandpiper/Delaware/1115/2022*	H11N2	99.31	100	EPI_ISL_16920544
