# Supplementary material for: Detection of H5N1-Related PB1 Sequences in a Low Pathogenic H11N2 Virus from South American Migratory Shorebirds
Source: Viruses. 2026 Jun 27;18(7):710. doi: 10.3390/v18070710 (PMC13431619; doi:10.3390/v18070710)
Supplement: Supplementary file 1 [file viruses-18-00710-s001.zip › viruses-4317979-Table S2.pdf]

Table S2: Comparison of Amino acid substitutions in the PB1 segment of representative H5N1 (HPAI) viruses relative to A/white_rumped_sandpiper/Brazil/PNLP_SM10/2023 (H11N2).

Virus Strain	Subtype	D3V	264	429	744	PB1-F2 (66S)
**A/white_rumped_sandpiper/Brazil/PNLP_SM10/2023**	**H11N2**	**-**	**E**	**L**	**L**	**-**
A/blue-winged_teal/Texas/GUI22-3250/2022	H5N1	-	D	K	M	-
A/chicken/Bolivar/CA-3500/2022	H5N1	•	E264D	K429R	L744M	•
A/red-shouldered_hawk/USA/WA-12226/2024	H5N1	•	E264D	K429R	L744M	•
A/skunk/AB/ABV-835/11/2022	H5N1	•	E264D	K429R	L744M	•
