# Supplementary figures and images for: Detection of H5N1-Related PB1 Sequences in a Low Pathogenic H11N2 Virus from South American Migratory Shorebirds
Source: Viruses. 2026 Jun 27;18(7):710. doi: 10.3390/v18070710 (PMC13431619; doi:10.3390/v18070710)

Tree scale: 0.1

PB2

- Antartica
- Eurasia
- America
- PNLP-Brazil

bootstrap

>70

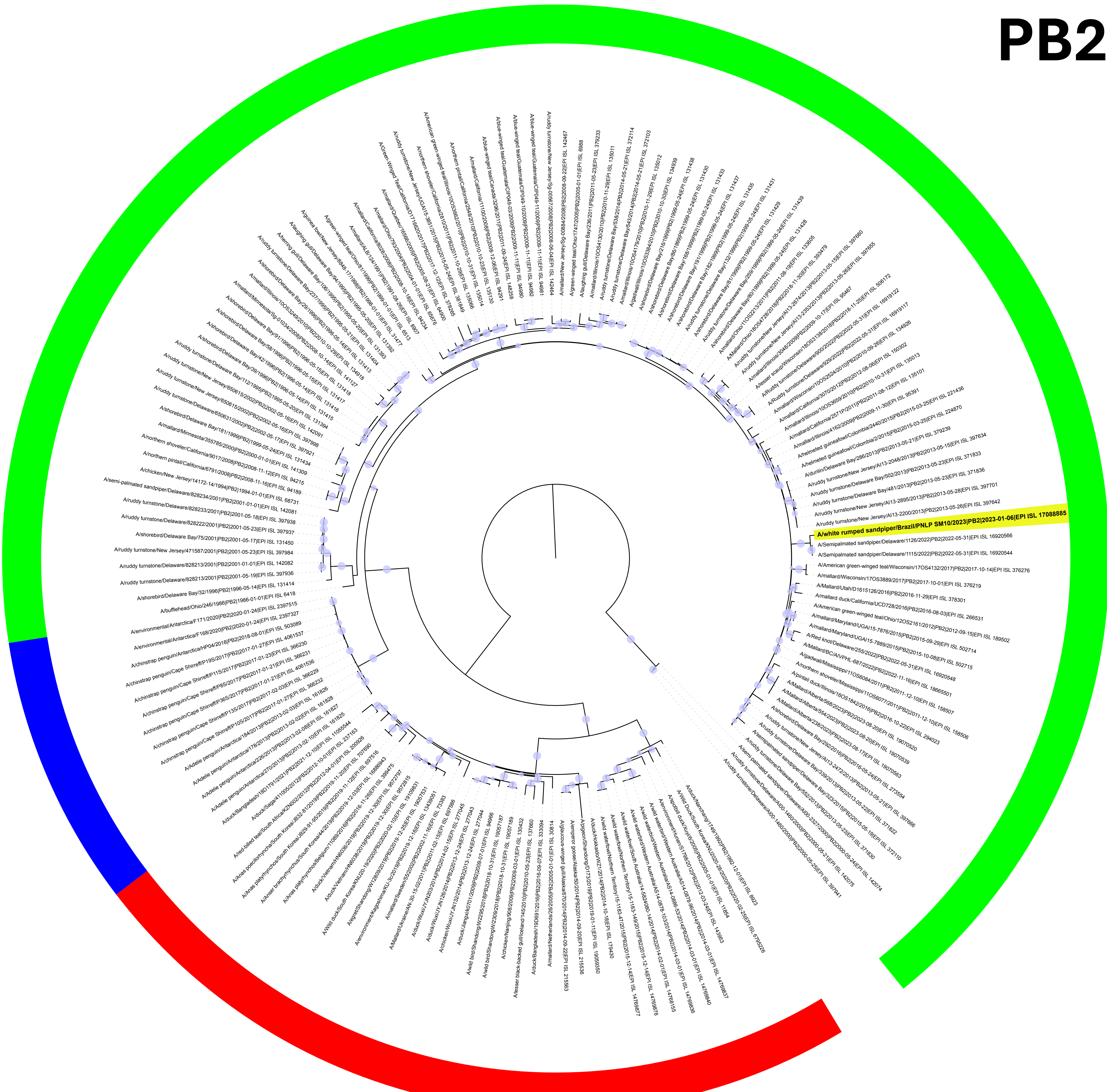

Supplement: Supplementary file 1 [file viruses-18-00710-s001.zip › viruses-4317979-Figure S1.pdf]

Tree scale: 0.1

# PA

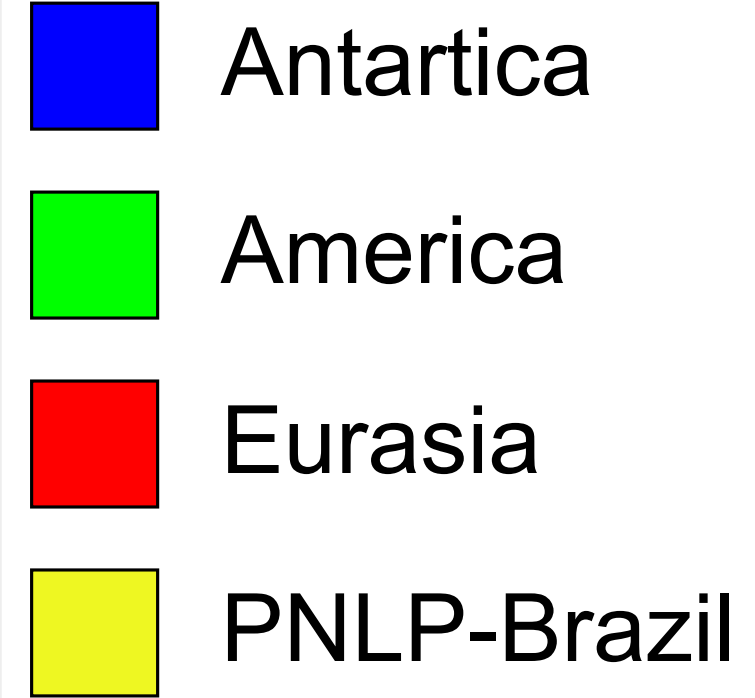

# bootstrap

● > 70

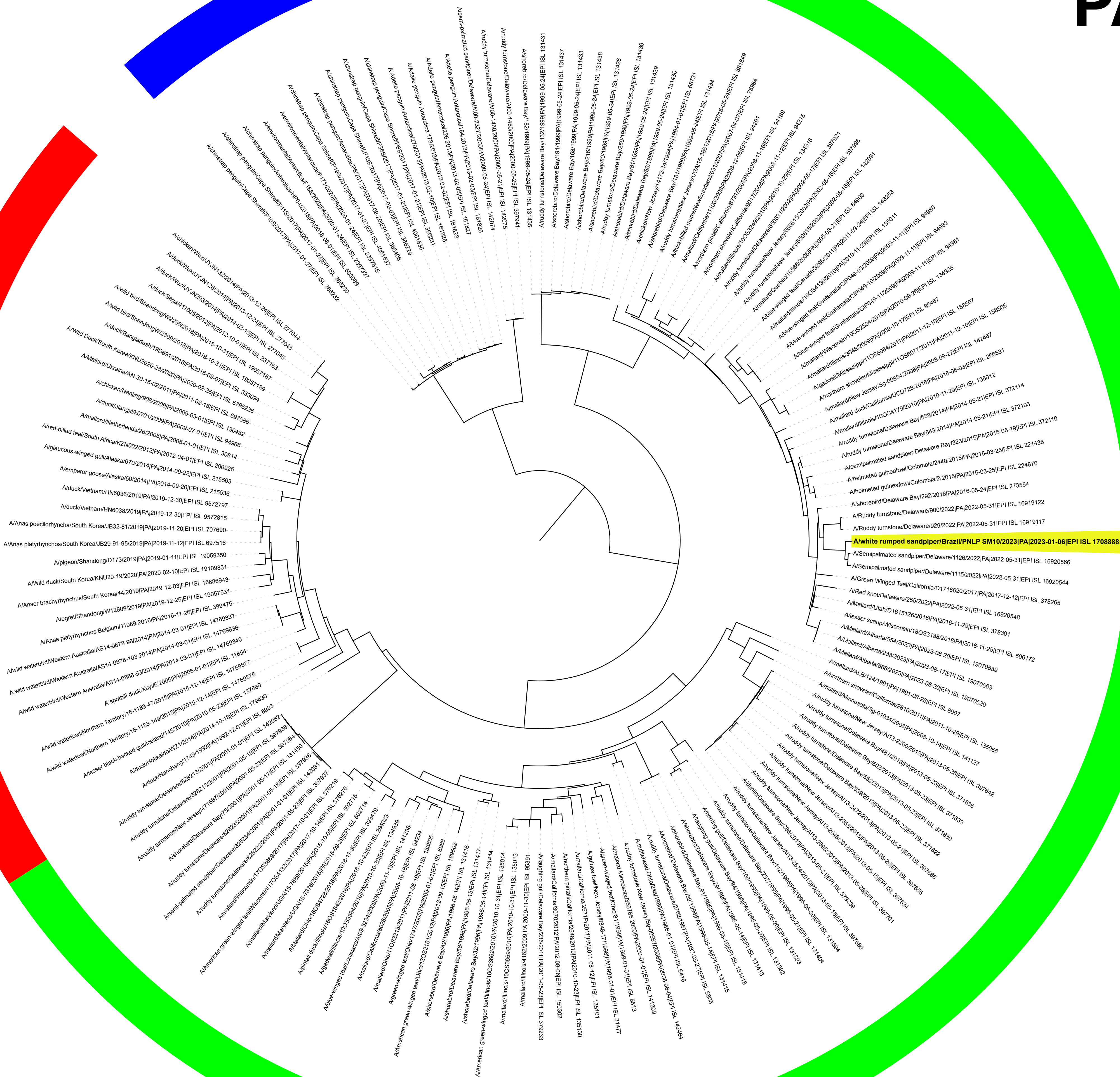

Supplement: Supplementary file 1 [file viruses-18-00710-s001.zip › viruses-4317979-Figure S2.pdf]

Tree scale: 0.1

NP

- Eurasia
- Antartica
- America
- PNLP-Brazil

bootstrap

> 70

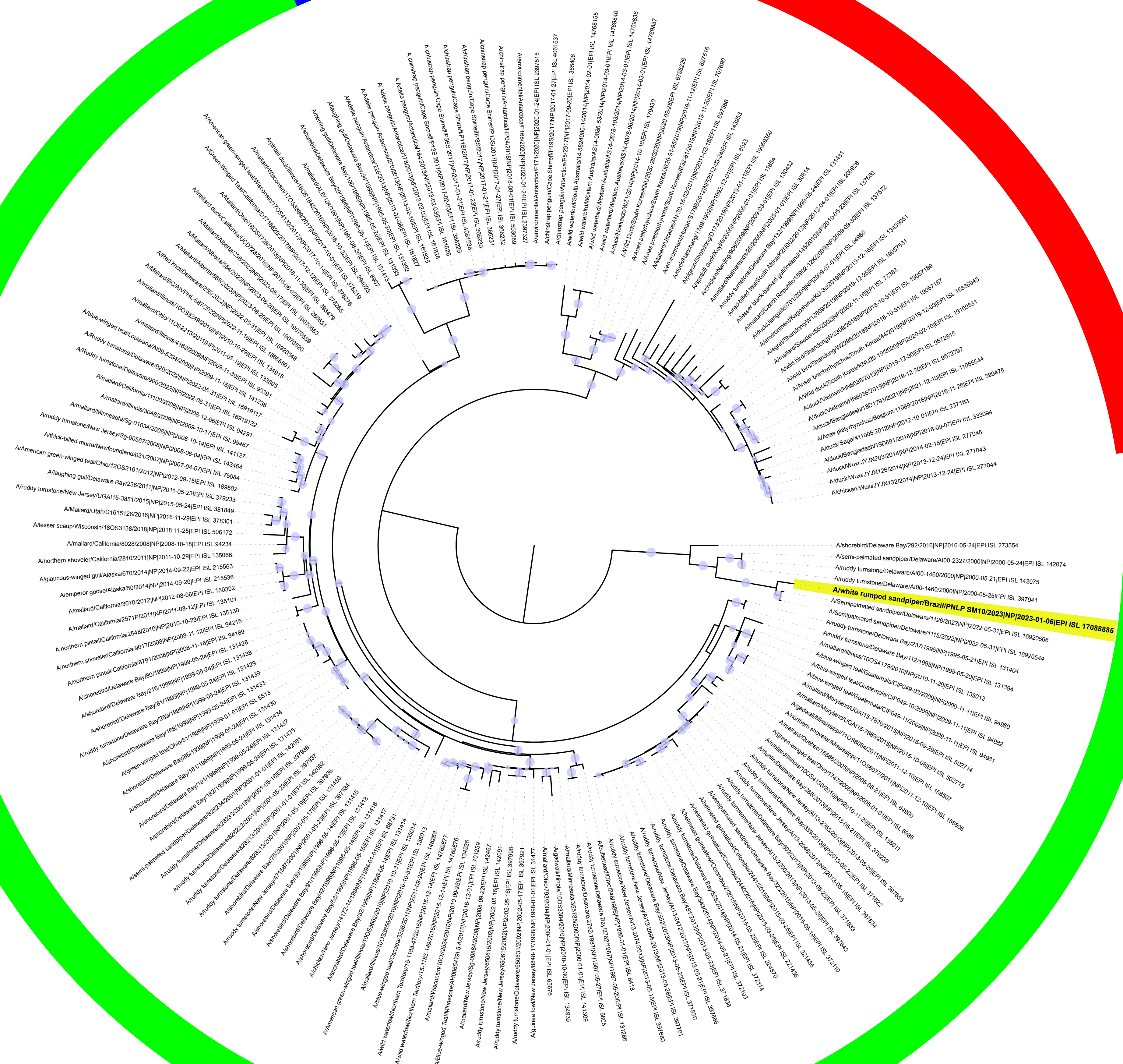

Supplement: Supplementary file 1 [file viruses-18-00710-s001.zip › viruses-4317979-Figure S3.pdf]

Tree scale: 0.1

**MP**

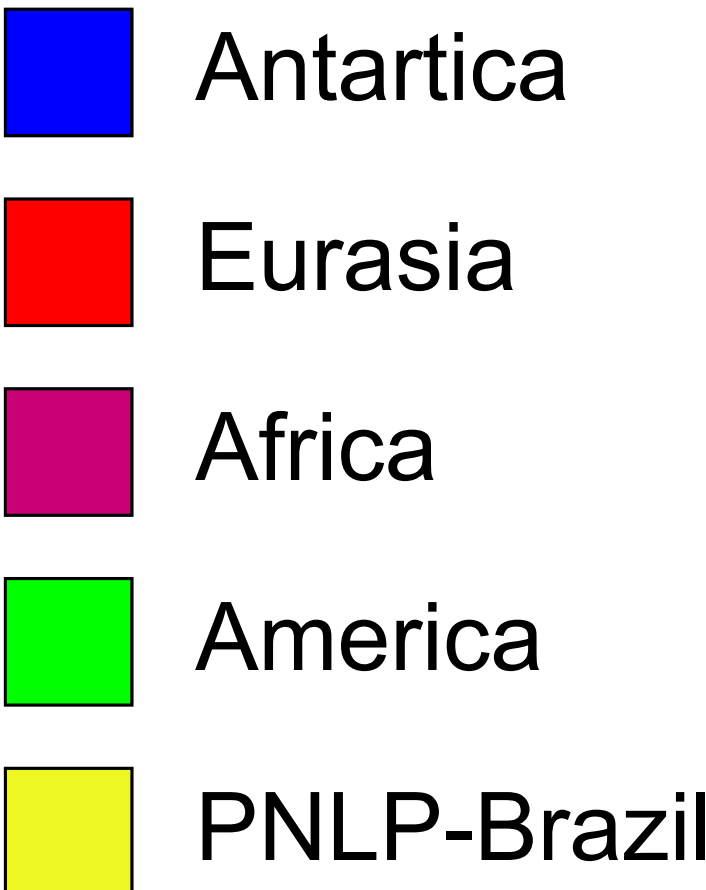

# bootstrap

● > 70

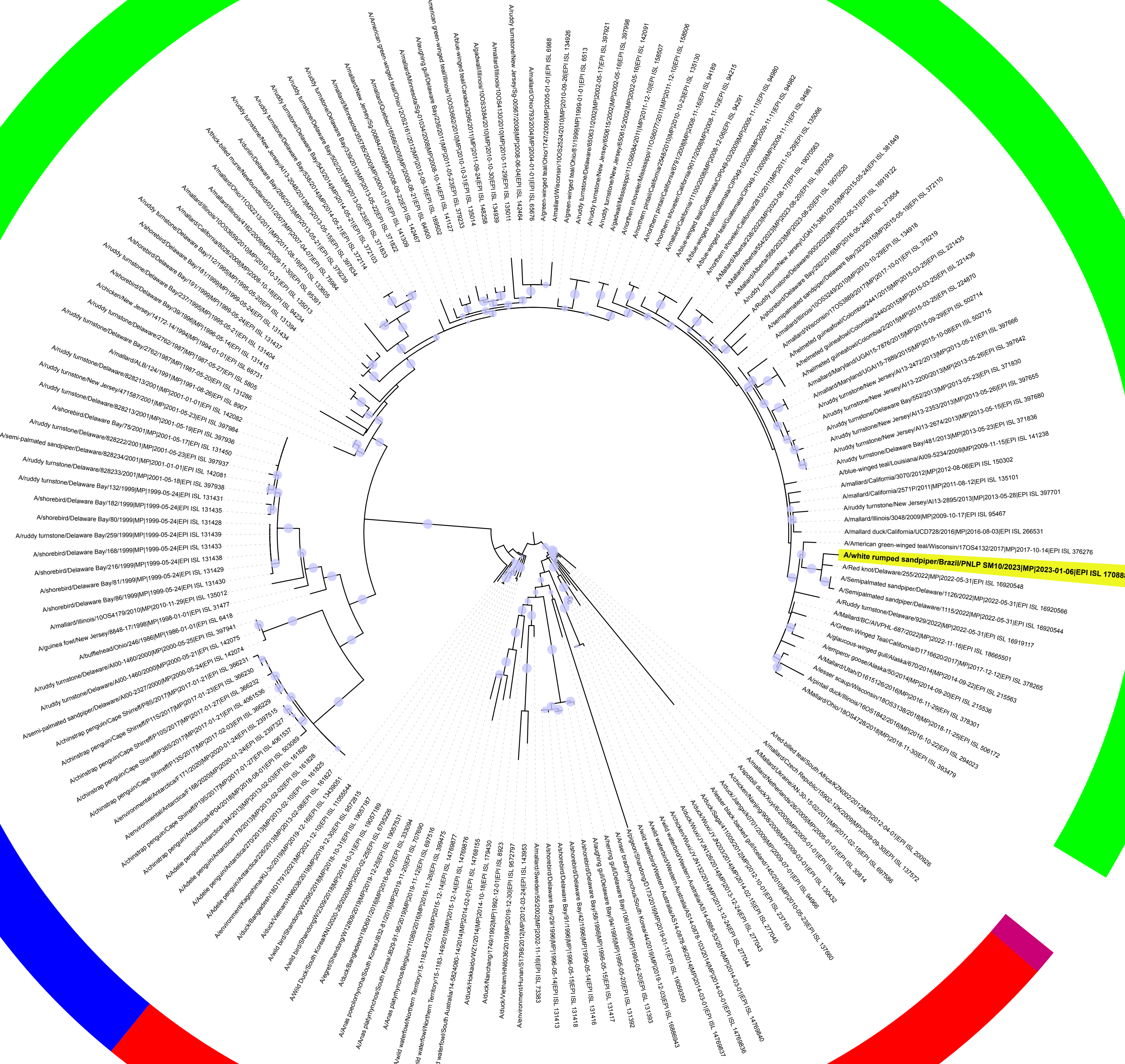

Supplement: Supplementary file 1 [file viruses-18-00710-s001.zip › viruses-4317979-Figure S4.pdf]

Tree scale: 1

NS

- Antartica
- Eurasia
- America
- PNLP-Brazil

bootstrap

> 70

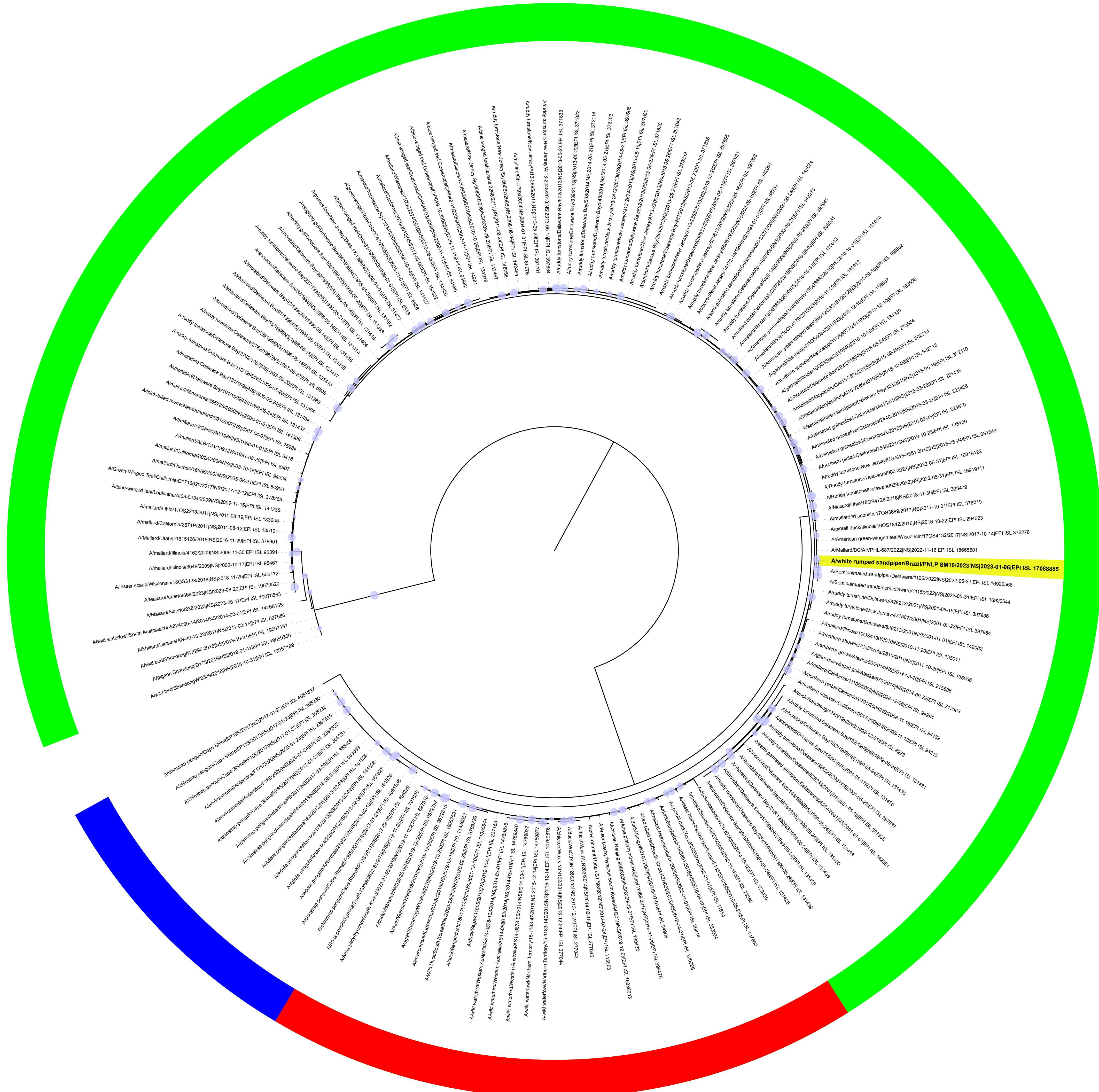

Supplement: Supplementary file 1 [file viruses-18-00710-s001.zip › viruses-4317979-Figure S5.pdf]
